# Chemoresistance to Cancer Treatment: Benzo-α-Pyrene as Friend or Foe?

**DOI:** 10.3390/molecules23040930

**Published:** 2018-04-17

**Authors:** Kevin Dzobo, Naseeha Hassen, Dimakatso Alice Senthebane, Nicholas Ekow Thomford, Arielle Rowe, Hendrina Shipanga, Ambroise Wonkam, M. Iqbal Parker, Shaheen Mowla, Collet Dandara

**Affiliations:** 1 International Centre for Genetic Engineering and Biotechnology (ICGEB), Cape Town Component, Wernher and Beit Building (South), University of Cape Town Medical Campus, Anzio Road, Observatory 7925, Cape Town, South Africa; SNTDIM001@myuct.ac.za (D.A.S.); arielle.rowe@icgeb.org (A.R.); hmshipanga@gmail.com (H.S.); 2 Division of Medical Biochemistry and Institute of Infectious Disease and Molecular Medicine, Faculty of Health Sciences, University of Cape Town, Anzio Road, Observatory 7925, Cape Town, South Africa; iqbal.parker@uct.ac.za; 3 Pharmacogenomics and Drug Metabolism Group, Division of Human Genetics, Department of Pathology, Faculty of Health Sciences, University of Cape Town, Anzio Road, Observatory 7925, Cape Town, South Africa; naseeha.hassen@gmail.com (N.H.); nicholas.thomford@uct.ac.za (N.E.T.); ambroise.wonkam@uct.ac.za (A.W.); collet.dandara@uct.ac.za (C.D.); 4 Division of Haematology, Department of Pathology, Faculty of Health Sciences, University of Cape Town, Anzio Road, Observatory 7925, Cape Town, South Africa; shaheen.mowla@uct.ac.za

**Keywords:** environmental pollution, benzo-α-pyrene, procarcinogen, esophageal cancer, cisplatin, 5-fluorouracil, paclitaxel, chemoresistance, drug metabolizing enzymes, apoptosis

## Abstract

**Background:** Environmental pollution such as exposure to pro-carcinogens including benzo-α-pyrene is becoming a major problem globally. Moreover, the effects of benzo-α-pyrene (BaP) on drug pharmacokinetics, pharmacodynamics, and drug resistance warrant further investigation, especially in cancer outpatient chemotherapy where exposure to environmental pollutants might occur. **Method:** We report here on the effects of benzo-α-pyrene on esophageal cancer cells in vitro, alone, or in combination with chemotherapeutic drugs cisplatin, 5-flurouracil, or paclitaxel. As the study endpoints, we employed expression of proteins involved in cell proliferation, drug metabolism, apoptosis, cell cycle analysis, colony formation, migration, and signaling cascades in the WHCO1 esophageal cancer cell line after 24 h of treatment. **Results:** Benzo-α-pyrene had no significant effect on WHCO1 cancer cell proliferation but reversed the effect of chemotherapeutic drugs by reducing drug-induced cell death and apoptosis by 30–40% compared to drug-treated cells. The three drugs significantly reduced WHCO1 cell migration by 40–50% compared to control and BaP-treated cells. Combined exposure to drugs was associated with significantly increased apoptosis and reduced colony formation. Evaluation of survival signaling cascades showed that although the MEK-ERK and Akt pathways were activated in the presence of drugs, BaP was a stronger activator of the MEK-ERK and Akt pathways than the drugs. **Conclusion:** The present study suggest that BaP can reverse the effects of drugs on cancer cells via the activation of survival signaling pathways and upregulation of anti-apoptotic proteins such as Bcl-2 and Bcl-xL. Our data show that BaP contribute to the development of chemoresistant cancer cells.

## 1. Introduction

Globally, environmental pollution is increasing, resulting in increased atmospheric levels of pollutants such as members of the polycyclic aromatic hydrocarbon family. The polycyclic aromatic hydrocarbon family contains hundreds of chemicals, of which benzo-α-pyrene (BaP) is the most studied due to its high levels in the environment and toxicity [1,2]. Sources of BaP include natural events such as the eruption of volcanoes, forest fires, and other sources such as exhaust fumes from cars and buses, cigarette smoke, and fried or roasted foods [3,4,5,6,7,8,9]. Areas that experience high volumes of vehicles such as cities and towns have higher levels of BaP in the environment than rural areas [10,11,12,13]. On average, people are exposed to about 20–800 ng of BaP per day [14,15,16,17]. Other estimates are much higher for example in the United States the estimated daily exposure amount is 2.2 µg per day [18]. BaP is a well-characterized procarcinogen [19,20,21,22,23] that is converted to reactive metabolites that can bind to nucleic acids and proteins and can result in uncontrolled proliferation of the cells [24,25,26]. Since the oesophagus is exposed to BaP ingested with food and water [27,28,29,30], we considered it relevant to evaluate the influence of BaP on esophageal cancer cell response to chemotherapeutic drugs.

One of the least studied yet very aggressive cancers is esophageal cancer [31,32,33,34]. Although it is the 8th most common type of cancer, it is the 6th leading cancer in terms of cancer deaths [32,33,34]. Esophageal squamous cell carcinoma (ESCC) is the predominant histological cancer type in Africa, Asia, and Latin America. ESCC is usually detected in its advanced stages. With a five-year survival rate of roughly 10–20%, ESCC is one of the deadliest cancers known [35,36]. Chemotherapy with cytotoxic drugs remains the bedrock of cancer treatment including ESCC. Several chemotherapeutic drugs have been approved for the treatment of ESCC and these include cisplatin, 5-flurouracil, and paclitaxel. Chemotherapy is given in cycles, usually a 2–4-week period, followed by a resting period to give the body enough time to recover. Patients normally undergo several cycles during treatment. Unless the case is serious, most patients are treated as outpatients. During this treatment period, patients are exposed to further environmental insults including chemicals in air, ingested food, and the water they drink. Few, if any, studies have ever investigated the influence of environmental pollutants on the response of cancer patients to chemotherapy. The mechanism(s) by which cancer cells develop chemoresistance is debatable with many reports implicating cancer stem cells and the tumor microenvironment [37,38,39,40,41].

This study focused on three chemotherapeutic drugs, namely cisplatin, 5-flurouracil, and paclitaxel, commonly used in the treatment of a wide range of malignancies, including breast, gastrointestinal, esophageal, cervical, skin, and lung cancer [42,43,44]. Combinations of these drugs, together with radiotherapy is the gold standard in the treatment of esophageal cancer [45,46]. The influence of environmental pollutants such as BaP on the response of esophageal cancer cells to combinations of these drugs has not been investigated before. Thus, the aim of the study was to investigate the influence of BaP on the response of WHCO1 esophageal cancer cells to chemotherapeutic drugs, cisplatin, 5-fluorouracil, or paclitaxel in vitro.

## 2. Results

### 2.1. Benzo-α-Pyrene Reverse Individual Drug-Induced Cell Death and Aapoptosis in WHCO1 Cells

**Figure 1:**
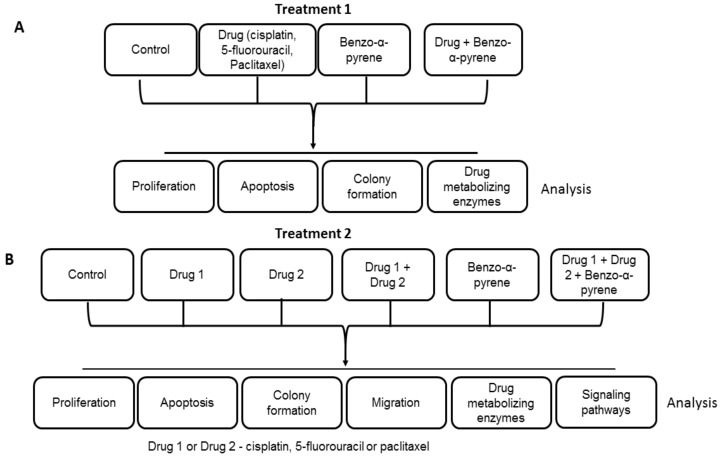
Scheme of WHCO1 and WHCO5 cancer cell treatment (**A**) Evaluation of effect of benzo-α-pyrene (BaP) on the response of WHCO1 and WHCO5 cancer cells to individual drugs; (**B**) Evaluation of effect of BaP on the response of WHCO1 and WHCO5 cancer cells to combinations of drugs.

The first step was to evaluate the effect of BaP on the response of WHCO1 cancer cells to individual drugs followed by the evaluation of BaP effect on the response of WHCO1 cancer cells to combinations of drugs (Figure 1A,B). The IC_50_ of cisplatin, measured via the MTT assay, was 18.5 ± 6.4 µM whilst that of 5-fluorouracil was 14.1 ± 3.8 µM. Both IC_50_ values were measured in WHCO1 cells over 24 h. The IC_50_ was determined as the concentration of drug needed to kill 50% of cells over 24 h. Similar IC_50_ values have been obtained before [47,48,49,50,51,52,53,54]. Our preliminary experiments showed that the use of concentrations above 10 µM would result in considerable cell death, and we used concentrations less than half the IC_50_ value (Supplementary Figure S1A–C). Paclitaxel IC_50_ is reported to be much lower than that of cisplatin and 5-fluorouracil [55,56,57]. We used 3.5 µM 5-fluorouracil, 4.2 µM cisplatin, and 2 µM for paclitaxel. The concentration for benzo-α-pyrene (10 µM) was based on our cell viability assays and from the literature value [58,59]. No morphological changes were observed in WHCO1 cells treated with 0.1% DMSO and 10 µM BaP alone (data not shown). Cell viability assays using different doses of BaP, from 0 µM to as high as 120 µM showed that the viability of WHCO1 cells decreased when the concentration of BaP was above 80 µM after 24 h of incubation (Supplementary Figure S1D). Two different methods were used to determine the effects of cisplatin, 5-fluorouracil, paclitaxel, and BaP on WHCO1 cell proliferation. Immunoblot analysis of proliferation markers, Ki67 and PCNA, showed that at early time points in the experiment there is a differential response to the presence of cisplatin, 5-fluorouracil, and paclitaxel (Supplementary Figure S2A,C,E). At 24 h after the start of the experiment, cisplatin, 5-fluorouracil, and paclitaxel caused downregulation of both Ki67 and PCNA. This downregulation was reversed in the presence of BaP, showing that BaP can increase WHCO1 cancer cell proliferation (Supplementary Figure S2A,C,E). Cell counting assays substantiated our immunoblot findings (Supplementary Figure S2B,D,F). Similar cell counting results were obtained using another esophageal cancer cell line, WHCO5 (Supplementary Figure S3A–C).

**Figure 2:**
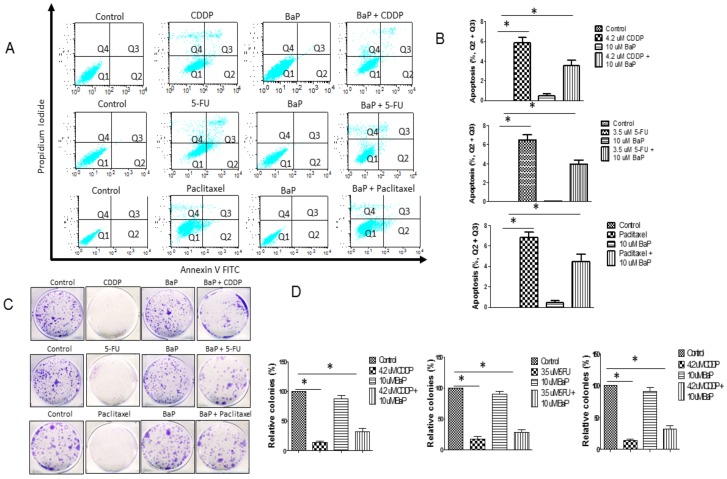
Benzo-α-pyrene abrogates drug-induced-apoptosis and colony formation inhibition. (**A**) WHCO1 cells (3 × 10^5^) were plated in 6-well plates overnight. WHCO1 cells were then treated with 0.1% DMSO, 4.2 µM CDDP, 3.5 µM 5-FU, 2 µM paclitaxel, and 10 µM BaP for 24 h. Cells were double stained with Annexin V and Propidium Iodide and analyzed by flow cytometry to detect apoptosis; (**B**) Quantification of apoptosis after treatment of WHCO1 cells as described in (**A**) based on the percentage of cells in each quadrant (Q1, Q2, Q3, Q4); (**C**) WHCO1 cells (1 × 10^3^) were plated in 6-well plates overnight. WHCO1 cells were then treated with 0.1% DMSO, 4.2 µM CDDP, 3.5 µM 5-FU, 2 µM paclitaxel, and 10 µM BaP and incubated for 8 days. After 8 days, colonies were fixed with 4% para-formaldehyde, stained with 0.1% crystal violet, and counted; (**D**) Quantification of colonies after treatment of WHCO1 cells as described in (**C**). * *p* < 0.05.

Annexin V and propidium iodide double staining confirmed the induction of apoptosis in the presence of cisplatin, 5-fluorouracil, and paclitaxel (Figure 2A,B, early apoptosis and late apoptosis shown in Q2 and Q3). Indeed, apoptotic cell death was also characterized by cytoplasmic blebbing (data not shown). BaP on its own did not cause any apoptosis. BaP, when combined with either cisplatin, 5-fluorouracil, or paclitaxel, seems to confer protection against apoptosis, as the percentage of cells undergoing apoptosis decreased. Cisplatin, 5-fluorouracil, and paclitaxel reduced colony formation significantly and BaP reverses the effect of each individual drug (Figure 2C,D). Drug-treated cells displayed significantly smaller and fewer colonies than control cells and those treated with BaP (Figure 2C,D). BaP in combination with drugs reduced drug-induced apoptosis and ameliorated the reduction in colony formation (Figure 2C,D). Again, using a different esophageal cancer cell line, WHCO5, we obtained similar apoptosis and colony formation results (Supplementary Figure S4A–D). Since results for the two esophageal cancer cell lines, WHCO1 and WHCO5, were similar, only WHCO1 cells were used in subsequent studies.

### 2.2. Cisplatin, 5-Fluorouracil, and Paclitaxel Differentially Affected the Expression of CYP1A1, CYP1A2, CYP1B1, and GSTP1 in WHCO1 Ccells

**Figure 3:**
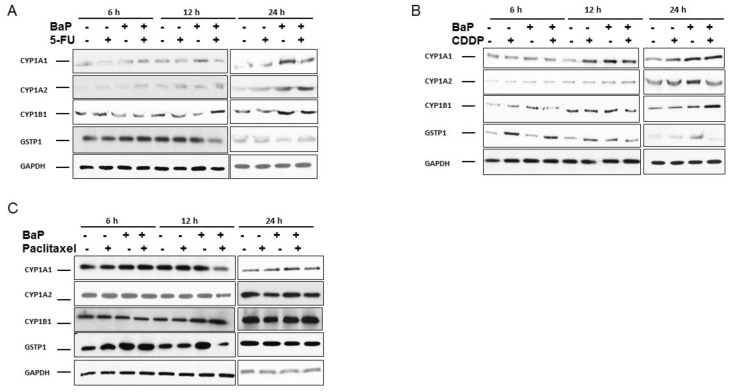
Benzo-α-pyrene differentially influence the expression of CYP1A1, CYP1A2, CYP1B1, and GSTP1 in WHCO1 in response to chemotherapeutic drugs. WHCO1 cells (5 × 10^5^) were plated in 6-well plates overnight. WHCO1 cells were then treated with 0.1% DMSO, 3.5 µM 5-FU, 4.2 µM cisplatin, 2 µM paclitaxel, and 10 µM BaP for 6, 12, and 24 h. Cells were lysed with RIPA buffer and proteins quantified using the BCA protein quantification assay. (**A**) Immunoblot analysis of proteins extracted from WHCO1 cells treated with 5-FU and BaP using anti-CYP1A1, CYP1A2, CYP1B1, and GSTP1 antibodies; (**B**) Immunoblot analysis of proteins extracted from WHCO1 cells treated with cisplatin and BaP using anti-CYP1A1, CYP1A2, CYP1B1, and GSTP1 antibodies; (**C**) Immunoblot analysis of proteins extracted from WHCO1 cells treated with paclitaxel and BaP using anti-CYP1A1, CYP1A2, CYP1B1, and GSTP1 antibodies. GAPDH was used as a loading control.

CYPs are members of the xenobiotic metabolizing enzymes involved in drug metabolism. We evaluated how the presence of cisplatin, 5-fluorouracil, paclitaxel, and BaP would affect the expression of four of these enzymes. At 6 h of incubation, BaP did not affect CYP1A1 protein levels. At 12 h and 24 h, however, the presence of BaP caused significant increases in CYP1A1 protein levels (Figure 3A). The treatment of WHCO1 cells with 5-fluorouracil and BaP resulted in a significant increase in CYP1A2 protein levels especially after 24 h (Figure 3A). 5FU caused differential *CYP1B1* gene expression in the presence of BaP at 6 and 12 h of incubation. After 24 h, BaP induced a significant increase in CYP1B1 protein levels (Figure 3A).

Cisplatin-treated cells showed significant increase in CYP1A1 protein levels only after 12 h of incubation (Figure 3B). The use of both cisplatin and BaP resulted in a significant increase in CYP1A1 and CYP1B1, higher than when each is used separately, thus having a synergistic effect on C*YP1A1* and *CYP1B1* gene expression (Figure 3B). Cisplatin and BaP induced a significant upregulation of CYP1A2 protein levels only after 12 h of incubation (Figure 3B). The presence of cisplatin caused significant increases in GSTP1 protein levels at all time points during the experiment (Figure 3B). Paclitaxel-treated cells showed no change in CYP1A1 protein levels (Figure 3C). After 12 h of incubation with both paclitaxel and BaP, CYP1A1 protein levels decreased significantly. The same trend was observed in the expression of CYP1A2. There was a differential expression of GSTP1 in the presence of paclitaxel and BaP (Figure 3C). In summary, BaP is associated with increased *CYP1A1* and *CYP1A2* gene expression. These genes are involved in drug metabolism and their increased expression might result in reduced drug efficacy.

### 2.3. BaP Protects WHCO1 Cancer Cells from the Effects of Cisplatin, 5-fluorouracil, and Paclitaxel Combination Therapy

**Figure 4:**
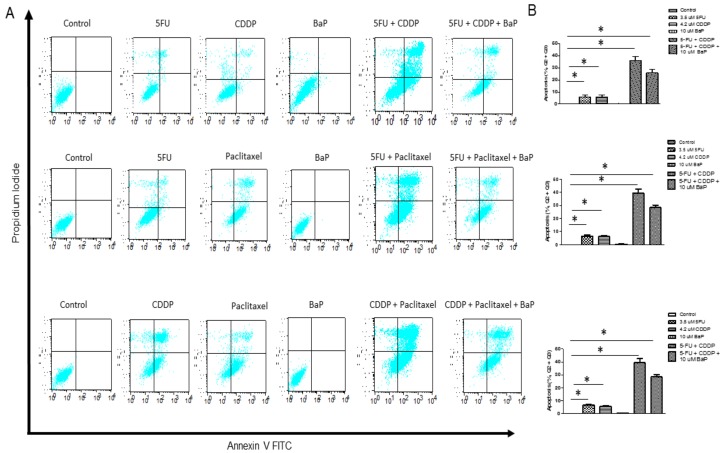
Benzo-α-pyrene reverses the dual effect of cisplatin, 5-FU, and paclitaxel on WHCO1 apoptosis. WHCO1 cells (5 × 10^5^) were plated in 6-well plates overnight. WHCO1 cells were then treated with 0.1% DMSO, 4.2 µM CDDP, 3.5 µM 5-FU, 2 µM paclitaxel, 10 µM BaP, and their combinations for 24 h. Cells were double stained with Annexin V and Propidium Iodide and analyzed by flow cytometry to detect apoptosis. (**A**) Flow cytometric analysis of WHCO1 cells after treatment with 0.1% DMSO, 4.2 µM cisplatin, 3.5 µM 5-FU, 2 µM, 10 µM BaP, and their combinations; (**B**) Quantification of apoptosis after treatment of WHCO1 cells as described in (**A**) based on the percentage of cells in each quadrant (Q1, Q2, Q3, Q4). * *p* < 0.05.

Chemotherapy is given as combinations of drugs and, to increase the relevance of our study, we evaluated the influence of BaP exposure on the response of WHCO1 esophageal cancer cells to combinations of chemotherapeutic drugs. As expected, drug-treated cells showed reduced proliferation compared to controls (Supplementary Figure S5A,B). A combination of cisplatin and 5-fluorouracil further reduced proliferation of WHCO1 cells compared to individual drugs (Supplementary Figure S5A,B). Similar results were obtained when WHCO1 cells were treated with 5-fluorouracil and paclitaxel (Supplementary Figure S5C,D) and a combination of cisplatin and paclitaxel reduced WHCO1 cell proliferation further compared to the effect of the individual drugs (Supplementary Figure S5E,F). Treatment of WHCO1 cells with a combination of 5-fluorouracil and cisplatin induced increased apoptosis compared to the individual drugs (Figure 4A,B, top panel). BaP had a protective effect on WHCO1 cancer cells treated with cisplatin and 5-fluorouracil as exposure of cancer cells to drugs in addition to BaP reduced the percentage of cells undergoing apoptosis (Figure 4A,B, top panel). Indeed, similar results were obtained when combinations of 5-fluorouracil and paclitaxel (Figure 4A,B, middle panel), and cisplatin and paclitaxel were used (Figure 4A,B, lower panel). The presence of BaP reversed the increased apoptosis observed when combinations of cisplatin, 5-fluorouracil, and paclitaxel were used.

**Figure 5:**
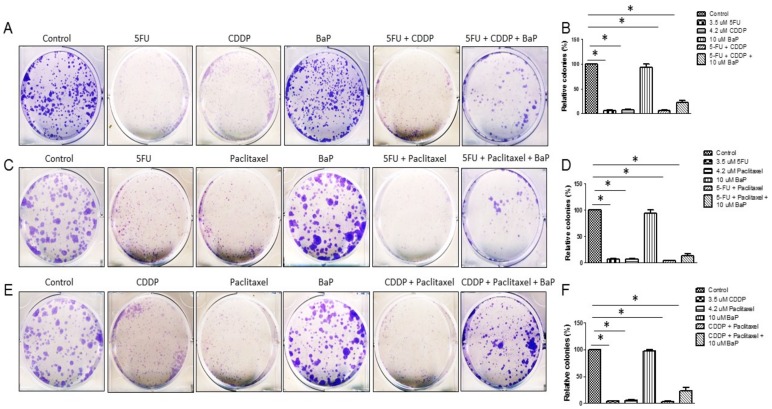
Benzo-α-pyrene reverses the dual effect of cisplatin, 5-FU, and paclitaxel on WHCO1 colony formation. WHCO1 cells (1 × 10^3^) were plated in 6-well plates overnight. WHCO1 cells were then treated with 0.1% DMSO, 4.2 µM CDDP, 3.5 µM 5-FU, 2 µM paclitaxel, 10 µM BaP, and their combinations. Cells were cultured for another 8 days. Para-formaldehyde (4%) was used to fix the cells and staining was done using 0.1% crystal violet. (**A**,**C**,**E**) Representative images of colonies formed when cells were treated with 5FU, CDDP, Paclitaxel, BaP and their combinations; (**B**,**D**,**F**) Colonies were counted using the UVP software and the relative numbers were plotted on a graph. * *p* < 0.05.

Commitment of cells to the apoptotic pathway depends on the balance between protein mediators of cell death (Bax included) and anti-apoptotic proteins such as Bcl-2 and Bcl-xL. As shown in Supplementary Figure S6, there is a drug-induced decrease in Bcl-2 and Bcl-xL mRNA levels. BaP alone caused an upregulation of Bcl-2 and Bcl-xL mRNA levels and reversed by 30–40% the effects of drugs combinations on the expression of the same genes (Supplementary Figure S6A–F). Drug-treated cells showed significantly reduced colony formation (Figure 5A–F). Combinations of cisplatin, 5-fluorouracil, and paclitaxel further reduced colony formation compared to individual drugs. BaP had no effect on WHCO1 cell colony forming abilities (Figure 5A–F). The presence of BaP reduced the effect of drugs combination on WHCO1 cell colony formation as larger and more colonies were formed compared to both individual and combinations of drugs (Figure 5A–F).

### 2.4. Effect of Combinations of Cisplatin, 5-Fluorouracil, Paclitaxel, and BaP on WHCO1 Cell Migration and Invasion

**Figure 6:**
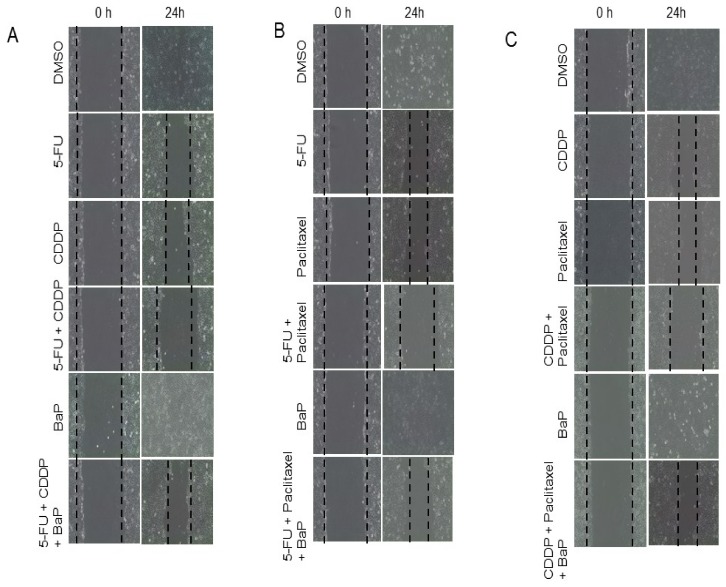
Benzo-α-pyrene abrogates the effect of cisplatin, 5-FU, paclitaxel, and their combinations on WHCO1 cell migration. WHCO1 cells (5 × 10^5^) were plated in 6-well plates until confluent. Scratch wounds were made using a 200 ul pipette tip and cells were treated with 0.1% DMSO, 4.2 µM CDDP, 3.5 µM 5-FU, 2 µM, 10 µM BaP, and their combinations 24 h. At indicated time points during incubation, images of the scratch wounds were taken using a Phase Contrast inverted microscope (Olympus CKX41). (**A**) Effect of BaP on WHCO1 cell migration in response to cisplatin, 5-FU, and their combinations; (**B**) Effect of BaP on WHCO1 cell migration in response to 5-FU, paclitaxel, and their combinations; (**C**) Effect of BaP on WHCO1 cell migration in response to cisplatin, paclitaxel, and their combinations. Results are shown as an average of three independent experiments.

To determine whether BaP would influence the effect of cisplatin, 5-fluorouracil, and paclitaxel on WHCO1 cell migration, we performed the wound-healing assay. The scratch wound assays showed that BaP-treated cells migrated at the same rate as control WHCO1 cells (Figure 6A–C; Supplementary Figure S7A–C). In contrast, drug-treated cells did not migrate much and the wound gap remained (Figure 6A–C; Supplementary Figure S7A–C). WHCO1 cells exposed to combinations of cisplatin, 5-fluorouracil, and paclitaxel migrated very little compared to controls. Combinations of cisplatin, 5-fluorouracil, and paclitaxel together with BaP resulted in WHCO1 cells migrating much further than combinations of drugs only (Figure 6A–C; Supplementary Figure S7A–C). The influence of BaP on WHCO1 cancer cell invasion in the presence of drugs was investigated using the trans-well assay. Matrigel invasion assays showed that same number of control cells and BaP-treated cells invaded through the Matrigel whilst drug combination-treated cells showed decreased invasion abilities compared to control cells (Supplementary Figure S7D–F). BaP reversed the effects of drugs on the invasive abilities of WHCO1 cells (Supplementary Figure S7D–F).

### 2.5. Chemotherapeutic Drugs Differentially Affect CYPs Expression and Survival Signaling Pathways

**Figure 7:**
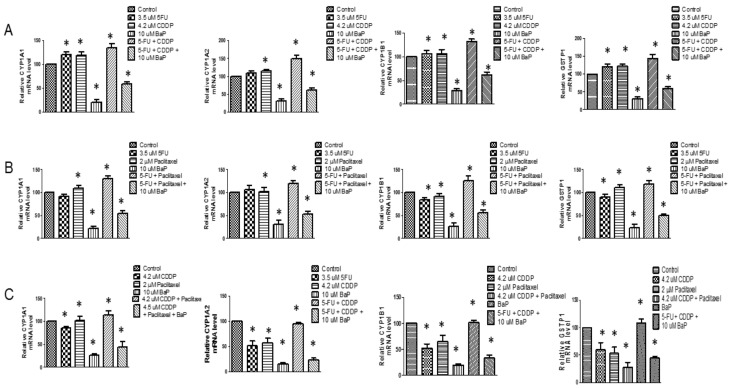
Benzo-α-pyrene differentially affect the expression of CYP1A1, CYP1A2, CYP1B1, and GSTP1 in WHCO1 cells in the presence of drugs. WHCO1 cells (5 × 10^5^) were plated in 6-well plates overnight. WHCO1 cells were then treated with 0.1% DMSO, 4.2 µM, 3.5 µM 5-FU, 2 µM paclitaxel, and 10 µM BaP for 24 h. (**A**) RT PCR analysis was performed using CYP1A1, CYP1A2, CYP1B1, and GSTP1 primers after treatment with a combination of cisplatin, 5-fluorouracil, and BaP; (**B**) RT PCR analysis was performed after treatment with a combination of 5-fluorouracil, paclitaxel, and BaP; (**C**) RT PCR analysis was performed after treatment with a combination of cisplatin, paclitaxel, and BaP. GAPDH was used as a normalizer. * *p* < 0.05.

The treatment of WHCO1 cells with combinations of cisplatin and 5-fluorouracil resulted in increased expression of CYP1A1 mRNA after 24 h (Figure 7A). Each individual drug also increased CYP1A1 mRNA expression (Figure 7A). BaP alone and in combinations with cisplatin and 5-fluorouracil caused significant increases in CYP1A1 mRNA levels (Figure 7A). The treatment of WHCO1 cells with cisplatin and 5-fluorouracil caused significant increases in CYP1B1 mRNA levels, while there were no major changes to the levels of CYP1A2 and GSTP1 mRNA levels (Figure 7A). A combination of 5-fluorouracil and paclitaxel resulted in increased CYP1A1 and CYP1A2 mRNA levels (Figure 7B), with an even higher increase when 5-fluorouracil, paclitaxel, and BaP were combined. There were changes to CYP1B1 and GSTP1 mRNA levels (Figure 7B). Combinations of cisplatin and paclitaxel caused significant increases in CYP1A1, CYP1A2, and CYP1B1 mRNA levels, but adding BaP to this mixture did not result in any further increase in the expression of these genes. There was however an increase in GSTP1 when BaP was added to the combination of cisplatin and paclitaxel (Figure 7C).

**Figure 8:**
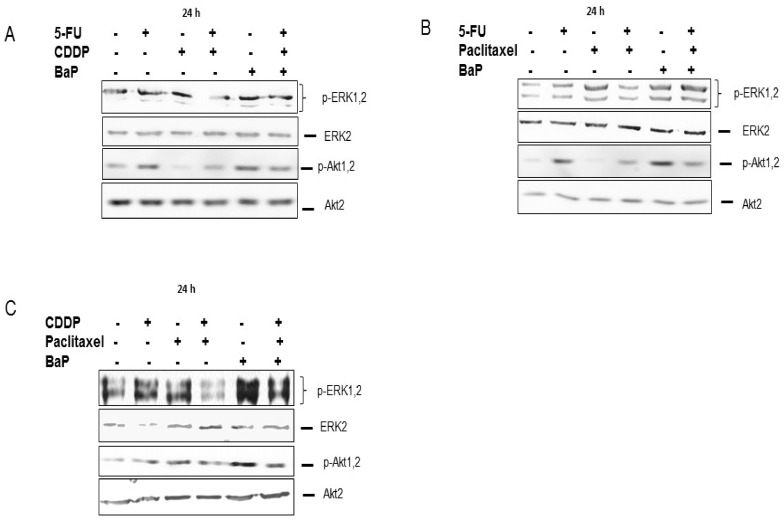
Benzo-α-pyrene reverse the effect of drugs on Akt and MEK-ERK signaling pathways in WHCO1 cells. WHCO1 cells (5 × 10^5^) were plated in 6-well plates overnight. WHCO1 cells were then treated with 0.1% DMSO, 4.2 µM cisplatin, 3.5 µM 5-FU, 2 µM, and 10 µM BaP for 24 h. Cells were lysed with RIPA buffer and proteins quantified using the BCA protein quantification assay. (**A**) Immunoblot analysis was performed using anti-p-ERK 1, 2, anti-p-Akt 1, 2, anti-ERK2, and anti-Akt2 antibodies after treatment with cisplatin, 5-fluorouracil, and BaP; (**B**) Immunoblot analysis was performed using anti-p-ERK 1, 2, anti-p-Akt 1, 2, anti-ERK2, and anti-Akt2 antibodies after treatment with 5-fluorouracil, paclitaxel, and BaP; (**C**) Immunoblot analysis was performed using anti-p-ERK 1, 2, anti-p-Akt 1, 2, anti-ERK2, and anti-Akt2 antibodies after treatment with cisplatin, paclitaxel, and BaP.

Signal transduction pathways including the Akt and MEK-ERK, involved in cancer cell survival, can be activated by chemotherapeutic drugs and can affect cell proliferation, gene expression, and invasion. Immunoblot analysis showed that, individually, cisplatin, 5-fluorouracil, and paclitaxel activated the MEK-ERK pathway while combination-treated cells showed a downregulation of the MEK-ERK signaling pathway, probably due to the toxic effects of the two drugs (Figure 8A–C). The 5-fluorouracil and paclitaxel-treated cells showed upregulation of the p-Akt (Figure 8C). BaP on the other hand significantly activated the MEK-ERK pathway rather than the Akt pathway (Figure 8A–C). No major changes were detected in the activation levels of the PI3K/Akt pathway in the presence of cisplatin (Figure 8A,C). Importantly, BaP reversed the downregulation of p-ERK 1, 2 induced by drug combinations (Figure 8A–C). In summary, BaP protect cancer cells from the effect of drugs through activating survival pathways such as the MEK-ERK and PI3K/Akt pathways.

## 3. Discussion

Globally, environmental pollution is increasing, resulting in increased atmospheric levels of chemicals such as BaP. Investigations into the role of BaP in carcinogenesis is well studied [20,21,60,61,62,63,64]. BaP contamination poses a serious health hazard to humans and those affected by diseases [25,65,66,67,68]. It is well documented that exposure to BaP and other chemicals found in soot, cigarette smoke, and exhaust fumes is associated with cancer in humans [5,6,7,8,9,58,69,70]. Due to its lipophilic nature, BaP can easily cross the cell membrane and accumulate in cells where it complexes with the aryl hydrocarbon receptor (AhR), stimulating its metabolism through induction of cytochrome P450 isoforms 1A1 (CYP1A1) and 1B1 (CYP1B1) [8,9,10].

People are exposed to different amounts of BaP depending on their daily activities. Ambient air contains BaP concentrations ranging from 20 mg/m^3^ to 100 mg/m^3^ [71]. Daily food consumption especially of foods such as fried chicken, smoked meat, and even potato chips contain higher levels of up to 125 ng/person/day [72]. The long half-life and the lipophilicity of BaP result in its accumulation in the human body to higher levels. Occupational exposure can result in very high levels of BaP accumulating in the human body. Limited studies have provided data on pollutants in rural areas [6]. In the rural areas the source of BaP would be burning wood for example. Concentrations of BaP depended on the season and ranged from 0.057 to 1.526 ng m^3^ in the cold season whilst it ranged from 0.009 to 0.111 ng m^3^ during the warm season in a study done in Croatia [6]. A major determinant of urban air genotoxicity is the fumes from traffic [73,74,75,76,77]. On average individual polycyclic aromatic hydrocarbons concentrations in ambient air in urban areas range from 1 to 30 ng/m^3^, with concentrations shooting up to 10–100 ng/cm3 being reported in road tunnels and large cities [78,79,80,81,82,83,84].

Cancer patients undergoing treatment such as chemotherapy are normally treated as outpatients. This exposes them to environmental insults such as BaP, especially smokers and passive smokers. Given that exposure to BaP, a well-characterized procarcinogen, is unavoidable, basic reasoning demands that the influence of such compounds on the efficacy of chemotherapeutic agents be investigated. The effect of BaP on patients undergoing chemotherapy has not been studied before. In addition, the in vitro effect of BaP on the response of WHCO1 esophageal cancer cells to the presence of two drugs studied at the same time hasn’t been investigated. To understand the toxicity of polycyclic aromatic hydrocarbons, an investigation of downstream genes including the CYP450 enzymes is important [85,86]. Being one of the most environmentally relevant polycyclic aromatic hydrocarbon, we used BaP as a reference to investigate the changes in WHCO1 cancer cell gene expression in response to the presence of chemotherapeutic agents in the presence of a pollutant.

Esophageal squamous cell carcinoma has a very low five-year survival rate [35,36] and the disease relapses and becomes resistant to most available chemotherapeutic drugs [35,36]. This poor prognosis emanates from the lack of efficacy of chemotherapeutic drugs and also the late detection of the disease. The anticancer drugs used in the study are the most common ones used in esophageal cancer and have different modes of actions. Firstly, cisplatin, also known as Platinol (CDDP), is an alkylating agent used for the treatment of many cancers such as esophageal, breast, cervical, and bladder cancer [87,88,89]. Secondly, 5-fluorouracil, an analogue of pyrimidine, is considered one of the most effective anticancer drug available [90,91]. Lastly, paclitaxel, also known as Taxol, is a member of the taxane family of several chemotherapeutic agents used to treat several cancers [92,93,94].

Different concentrations of these drugs have been used in several experimental setups [53,95,96,97,98,99,100]. Several studies have shown that the use of cisplatin and 5-fluorouracil combination is effective and safe in patients with advanced squamous cell oral cancer and head and neck carcinomas [101,102,103]. In addition, cisplatin and paclitaxel have been used to induce apoptosis in several cell lines including ovarian carcinoma cells [104,105,106]. A recent study evaluated the use of paclitaxel and 5-fluorouracil regimen to treat patients with advanced oesophageal carcinoma [107]. Whilst most of these studies show that it is safe and effective to use these combinations of drugs to treat the above mentioned cancers, none of these studies evaluated how such treatment regimens will be affected by the presence of polycyclic aromatic hydrocarbons such as BaP, making our study very relevant. A study to determine the toxicity of cisplatin as a treatment of malignant pleural effusions showed that a great variability of intrapleural cisplatin concentration depends on plasma diffusion [108]. Previous studies have shown that low concentrations of Paclitaxel such as 3 µM inhibited cell proliferation as effective as high concentrations [109,110]. Similar to our results, paclitaxel has been shown to cause G2 cell cycle arrest [109,110].

Our initial investigations, using individual drugs, showed that BaP reverses the effects of cisplatin, 5-fluorouracil, and paclitaxel on WHCO1 esophageal cancer cell proliferation by 20–30% compared to that of controls. Several studies have shown that BaP has proliferation-inducing properties. Dutta and colleagues found that BaP concentrations up to 80 µM did not cause any changes to glial cell viability after 48 h of BaP administration, compared to control cells [111]. Beside inducing proliferation and reversing the effects of cisplatin, 5-fluorouracil, and paclitaxel on WHCO1 cancer cell proliferation, BaP also reversed the effect of the same drugs on WHCO1 cancer cell apoptosis and colony formation. The observed decrease in cellular proliferation in the presence of drugs is expected as one of the modes of action of the drugs is inhibition of cellular growth and the killing of fast growing cells [112,113]. Contrary to our results, BaP has been found to induce apoptosis in several cells including neurons and hepatocytes [114,115,116,117].

In summary, our results show a differential expression of CYPs in WHCO1 cells in response to the presence of drugs with more upregulation than downregulation of genes. The precise mechanism responsible for the up-regulation caused by the drugs remains to be elucidated. Earlier studies have shown that 5-fluorouracil induces the activity of cyclin A-cyclin-dependent kinase 2 (Cdk2), an important cell cycle regulatory protein [118,119]. Interestingly, inhibition of the AhR has been shown to down-regulate cyclin and Cdk-2 expression [120,121,122]. Thus, it is possible that an up-regulation in Cdk2 activity, in this case caused by cisplatin and 5-fluorouracil, could be linked to a corresponding up-regulation in AhR activity ultimately resulting in increased *CYP1* expression [121,122]. Alternatively, 5-fluorouracil has also been shown to activate the NF-E2 p45-regulated factor (Nrf2) signaling pathway. The regulatory region of the *Nrf2* gene encompasses several AhR-binding response elements [123,124,125].

Several reports have shown that BaP has an effect on genes involved in cell cycle, migration, DNA repair, and apoptosis [126,127,128,129,130,131,132,133]. Proteins such as Bcl-2, Bax, and Bcl-xL are downstream of DNA damage and repair processes and the levels of these proteins determine whether cells undergo apoptosis or not [134]. Overall, our results show decreased Bcl-2 and Bcl-xL when cells were treated with drugs and even more decrease when drugs combinations were used. It is possible that this BaP-induced increase in Bcl-21 and Bcl-xL reverses drug-induced apoptosis. Several reports have shown that Bcl-2 and Bcl-xL are involved in the development of resistance to paclitaxel in cancer cells [135,136,137,138,139,140,141,142].

Several signaling pathways have been shown to be activated by the presence of reactive oxygen species, a by-product of cellular activation by BaP [111,143,144,145,146,147,148,149]. The PI3K-Akt signaling pathway is one of the most important pathways involved in the proliferation and survival of cells [150,151,152]. Our data show that indeed the MEK-ERK and PI3K-Akt signaling pathways are activated by both drugs and BaP, with BaP the stronger activator. The present study suggest that BaP can reverse the effects of drugs on cancer cells, including apoptosis, and this may be mediated by the activation of survival pathways such as MEK-ERK and Akt pathways in addition to upregulation of proteins such as Bcl-2 and Bcl-xL. This can result in resistant cancer cells, adding more weight to the growing concerns to the problem of environmental pollution.

## 4. Materials and Methods

### 4.1. Cell Culture and Treatments

WHCO1 and WHCO5 cells, derived from primary oesophageal squamous cell carcinoma in South Africa [153], were maintained in Dulbecco’s Modified Eagle Medium (DMEM) (Sigma-Aldrich, St. Louis, MO, USA) supplemented with 10% foetal bovine serum (FBS) (GIBCO, Invitrogen, Carlsbad, CA, USA), penicillin (100 U/mL), and streptomycin (100 µg/mL) (Sigma-Aldrich, St. Louis, MO, USA) at 37 °C in a humidified atmosphere of 5% CO_2_. These cells have been extensively used to study carcinogenesis and drug discovery [95,154]. WHCO1 and WHCO5 cells (5 × 10^5^ cells/well) were seeded in 6 cm dishes and allowed to attach. The cell culture media was replaced with fresh media prior to the start of treatment. Cell were treated by the addition of cisplatin (M_w_ 300.05; CAS 15663-27-1; Sigma, Marlborough, MA, USA), 5-fluorouracil (M_w_ 130.08 g/mol; CAS 51-21-8; Sigma), paclitaxel (M_w_ 853.91 g/mol; CAS 33069-62-4; Sigma), or BaP (M_w_ 252.31; CAS 50-32-8; Sigma) at the indicated concentrations for different time periods [53]. Drug concentrations at less than half of the IC_50_ were used in evaluating gene expression changes. The concentrations used were: 3.5 µM 5-fluorouracil; 4.2 µM cisplatin, 2 µM paclitaxel, 10 µM BaP, and combinations of 3.5 µM 5-fluorouracil, 4.2 µM cisplatin, 2 µM paclitaxel, and 10 µM BaP; and 0.1% DMSO (control). Incubation with drugs was done for 6, 12, and 24 h. For proliferation assays WHCO1 and WHCO5 cells were incubated for periods of up to 72 h.

### 4.2. Cell Viability Assay

Cell viability of WHCO1 and WHCO5 cancer cells after exposure to 0.1% DMSO (control), cisplatin, 5-flurouracil, paclitaxel, and BaP was determined by the MMT assay. The MTT reagent (3-[4,5-dimethylthiazol-2yl]-2,5-diphenyl-tetrazolium bromide) was first dissolved in phosphate-buffered saline (PBS) and stored at 20 °C in the freezer. WHCO1 and WHCO5 cancer cells (5 × 10^3^/well) were plated in 96-well plates and incubated overnight. Different concentrations of cisplatin, 5-flouorouracil, paclitaxel, or combinations, and 10 µM BaP were added to the media and cells were cultured for 24 h. The MTT reagent (20 µL) was added to each well and incubation continued for 4 h. After clearing the supernatant, DMSO was then added to each well and cells were shaken for 15 min. The resulting colour changes were read as optical density on a microplate reader. Cell viability was also determined by counting viable cells using the Countess Cell Counter. Assays were performed at the same time and done in triplicates. Results are reported as mean ± S.D. Experiments were repeated at least twice.

### 4.3. Microscopy

Cell morphology was observed and photographed using a light microscope (Olympus CKX41 with SC30 camera, Olympus Optical Co., Tokyo, Japan). Images were taken at 10X and 20X magnification. WHCO1 and WHCO5 cancer cells were treated as described above and observed after 24 h of incubation.

### 4.4. Annexin V Assay for Apoptosis

Apoptosis was evaluated through double staining with Annexin V and Propidium Iodide according to manufacturer’s instructions followed by flow cytometric analysis. WHCO1 and WHCO5 cells were incubated with cisplatin, 5-fluorouracil, paclitaxel, and BaP for 24 h and washed with PBS. Cells were harvested by trypsinisation using 0.05% trypsin-EDTA and resuspended in Annexin V binding buffer and stained with Annexin V conjugated to FITC and Propidium Iodide as described by the manufacturer’s instructions. Incubation in the dark was done for 15 min at 25 °C. The Beckman Coulter Flow Cytometer (Beckman Coulter, Life Sciences, Indianapolis, IN, USA) was used for flow cytometric analysis. Data acquisition was done on 2 × 10^4^ events per treatment condition. Analysis was performed using the Cellquest software (Version 5.1, Becton Dickinson, Franklin Lakes, NJ, USA).

### 4.5. Scratch Wound Assay

The effect of cisplatin, 5-fluorouracil, paclitaxel, and BaP on WHCO1 cell migration was evaluated by the wound healing assay. WHCO1 cells (1 × 10^6^) were cultured in 6-well plates and incubated overnight in DMEM containing 10% FBS. WHCO1 cells were cultured until confluent before the scratch wound assay was performed. Several lines were drawn underneath the dishes with a marker. These served as marks for the scratch wound experiment to be measured. Three parallel scratch wounds were made using a 200 µL (yellow) pipet tip. Washing was done twice with PBS to remove debris and fresh media was added. WHCO1 cells were then treated with either 0.1% DMSO, cisplatin, 5-fluorouracil, paclitaxel, and BaP for 24 h as indicated. Images were taken at 0 and at 24 h of culture. Scratch wounds were observed using phase contrast inverted microscopy on an inverted microscope (Olympus CKX41).

### 4.6. Transwell Invasion Assay

WHCO1 cells were cultured to 80% confluency before use in invasion assays. Matrigel (BD Biosciences, Bedford, MA, USA) was thawed overnight at 4 °C and then kept on ice. Transwell culture plates, with permeable cell culture inserts of 12 mm diameter and 8 µm pore size membranes (Corning Inc., Corning, NY, USA) were chilled to 4 °C and kept in the fridge. Matrigel (100 µL of 2 mg/mL) (BD Biosciences, Franklin Lakes, NJ, USA) was added to the upper chamber of the insert. The plates are then incubated at 37 °C for 2 h to solidify the Matrigel. WHCO1 cells were trypsinised and resuspended in DMEM. 1 × 10^5^ WHCO1 cells were added to the upper compartment and allowed to attach for 6 h before being treated with cisplatin, 5-fluorouracil, paclitaxel, or BaP. The bottom well contained media with 10% FBS as attractant, whilst the upper compartment of the insert contained media 1% FBS. WHCO1 cells were treated with 0.1% DMSO, drugs, and 10 µM BaP) and incubation was done for 24 h. At the end of the experiment the insert was treated as follows: cells and the Matrigel in the upper compartment were removed gently by wiping with a cotton swab. WHCO1 cells on the lower side of the insert membrane were fixed with 5% glutaraldehyde at room temperature for 10 min. Cells were then stained with 1% crystal violet in 2% ethanol at room temperature for 20 min. Inserts were washed three times and dried. The number of cells that invaded through the Matrigel were counted under the phase contrast microscope using the 10× objective. Four different fields were chosen and the average number of WHCO1 cells was obtained for each treatment. The invaded cell numbers were plotted onto a graph.

### 4.7. Quantitative RT-PCR Analysis

Total RNA was isolated using Qiazol (Roche, Berlin, Germany) as per the manufacturer’s instructions. cDNA was synthesized from 5 µg total RNA and used for quantitative real time reverse transcription polymerase chain reaction (RT PCR) analysis. RT PCR was carried out under the following conditions: 95 °C for 3 min (pre-incubation), followed by 40 cycles of 95 °C for 20 s, 62 °C for 20 s, and 72 °C for 20 s. The comparative critical threshold (Ct) method was used for the calculation of expression fold change between samples. Gene expression was calculated as fold induction, relative to the mean value of control samples using the equation 2^−∆∆Ct^. GAPDH was used as the housekeeping gene. Primers used are shown in Supplementary Table S1. Experiments were performed in triplicate.

### 4.8. Immunoblot Analysis

Cells were treated as described above and lysed in RIPA buffer and 1X complete protease inhibitor cocktail was added (Roche, Mannheim, Germany). The BCA Protein Quantification kit was used to quantify protein concentrations (Pierce, Rockford, IL, USA). Immunoblot analysis was performed using the following primary antibodies: rabbit anti-Ki67, goat anti-CYP1A1, rabbit anti-GAPDH, rabbit anti-PCNA, mouse CYP1A2, rabbit anti-CYP1B1, rabbit anti-GSTP1, rabbit anti-p-38 MAP kinase, anti-*p*-ERK 1, 2, anti-ERK 2, anti-*p*-Akt, and anti-Akt. The secondary antibodies used were goat anti-mouse IgG horseradish peroxidase (HRP) conjugate and goat anti-rabbit IgG HRP conjugate. The Lumiglo Chemiluminescent substrate (KPL, Gaithersburg, MD, USA) was used for the detection. All experiments were done in triplicates.

### 4.9. Colony Formation

WHCO1 cells were cultured overnight in 6-well plates at 500 cells per well. DMSO, cisplatin, 5-fluorouracil, paclitaxel, and BaP were added at the indicated concentrations and incubation was continued for 24 h. 0.1% DMSO was used for the control cells. Cells were then incubated in normal media for another 8 days. Methanol (100%) was used to fix the cells and cells were stained with 0.5% crystal violet. Colonies were counted using UVP software and the numbers were plotted on a graph. Images were taken using a camera. The experiments were performed at least three times.

### 4.10. Statistical Analysis

GraphPad Prism 5 (GraphPad Software Inc., San Diego, CA, USA) was used for statistical analysis to analyze the significance of the differences between the different treatment groups. To evaluate statistical significance between the different groups. One-way analysis of variance (ANOVA) was used and the Student’s *t* test was used to analyze differences between two independent groups. Data are shown as means ± SD. Values of *p <* 0.05 were considered significant. Experiments were performed at least in triplicate.

## Supplementary Materials

The following are available online, Supplementary Table S1: Oligonucleotide primer sequences used for RT-qPCR; Supplementary Figure S1: Inhibition rates of WHCO1 cancer cells incubated with different concentrations of cisplatin, 5fluorouracil, paclitaxel, and BaP for 24 h; Supplementary Figure S2: Benzo-α-pyrene reverse the effect of cisplatin, 5-FU, and paclitaxel on WHCO1 cell proliferation; Supplementary Figure S3: Benzo-α-pyrene reverse the effect of cisplatin, 5-FU, and paclitaxel on WHCO5 cell proliferation; Supplementary Figure S4: Benzo-α-pyrene abrogates drug-induced apoptosis and colony formation inhibition; Supplementary Figure S5: Benzo-α-pyrene reverse the dual effects of cisplatin, 5-FU, and paclitaxel on WHCO1 cell proliferation; Supplementary Figure S6: Benzo-α-pyrene upregulates Bcl-2 and Bcl-xL expression in WHCO1 cancer cells; Supplementary Figure S7: Benzo-α-pyrene abrogates the effect of cisplatin, 5-FU, paclitaxel, and their combinations on WHCO1 cell migration and invasion.
