# Supplementary material for: Chemoresistance to Cancer Treatment: Benzo-α-Pyrene as Friend or Foe?
Source: Molecules. 2018 Apr 17;23(4):930. doi: 10.3390/molecules23040930 (PMC6017867; doi:10.3390/molecules23040930)
Supplement: Supplementary file 1 [file molecules-23-00930-s001.pdf]

Supplementary Table S1. Oligonucleotide primer sequences used for RT-qPCR

Gene	Forward	Reverse
GAPDH	5-GCTCTCCAGAACATCATCC-3′	5′-GCCTGCTTCACCACCTTC-3′
BCL-2	5′-CTGCACCTGACGCCCTTCACC-3′	5′-CACATGACCCCACCGAACTCAAAGA-3′
BCL-xL	5′-GATCCCCATGGCAGCAGTAAAGCAAG-3′	5′-CCCCATCCCGGAAGAGTTCATTCACT-3′
CYP1A1	5′-TGGATGAGAACGCCAATGTC-3′	TGGGTTGACCCATAGCTTCT-3′
CYP1A2	5′-CACCCTGCCAATCTCAAGCAC-3′	AGAAGCTCTGTGGCCGAGAAGG-3′
CYP1B1	5′-CACTGCCAACACCTCTGTCTT-3′	CAAGGAGCTCCATGGACT-3′
GSTP1	5′-CCTGTACCAGTCCAATACCATCCT-3′	TCCTGCTGGTCCTTCCCATA-3′

Supplementary Figures

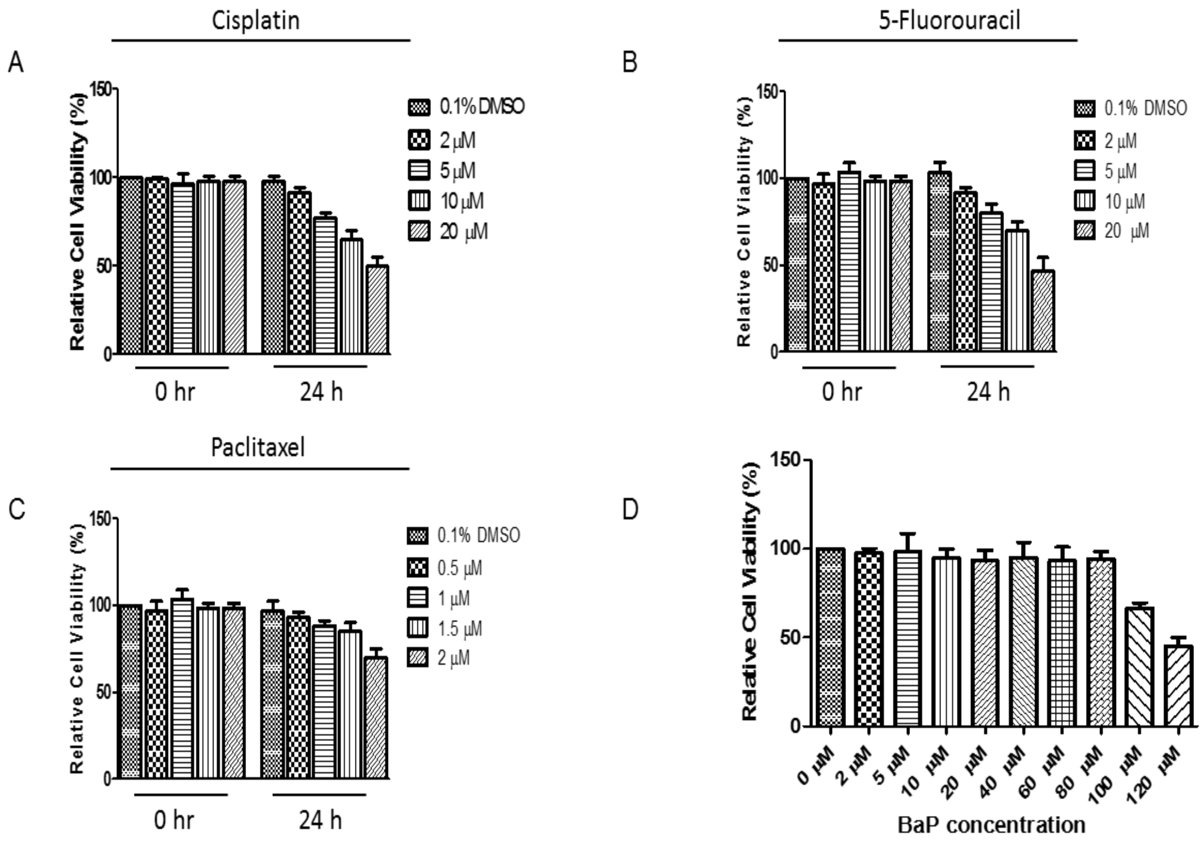

Supplementary Figure S1. (A) Inhibition rates of WHCO1 cancer cells incubated with different concentrations of cisplatin for 24 h. (B) Inhibition rates of WHCO1 cancer cells incubated with different concentrations of 5-fluorouracil for 24 h. (C) Inhibition rates of WHCO1 cancer cells incubated with different concentrations of paclitaxel for 24 h. (D) Inhibition rates of WHCO1 cancer cells incubated with different concentrations of BaP for 24 h.

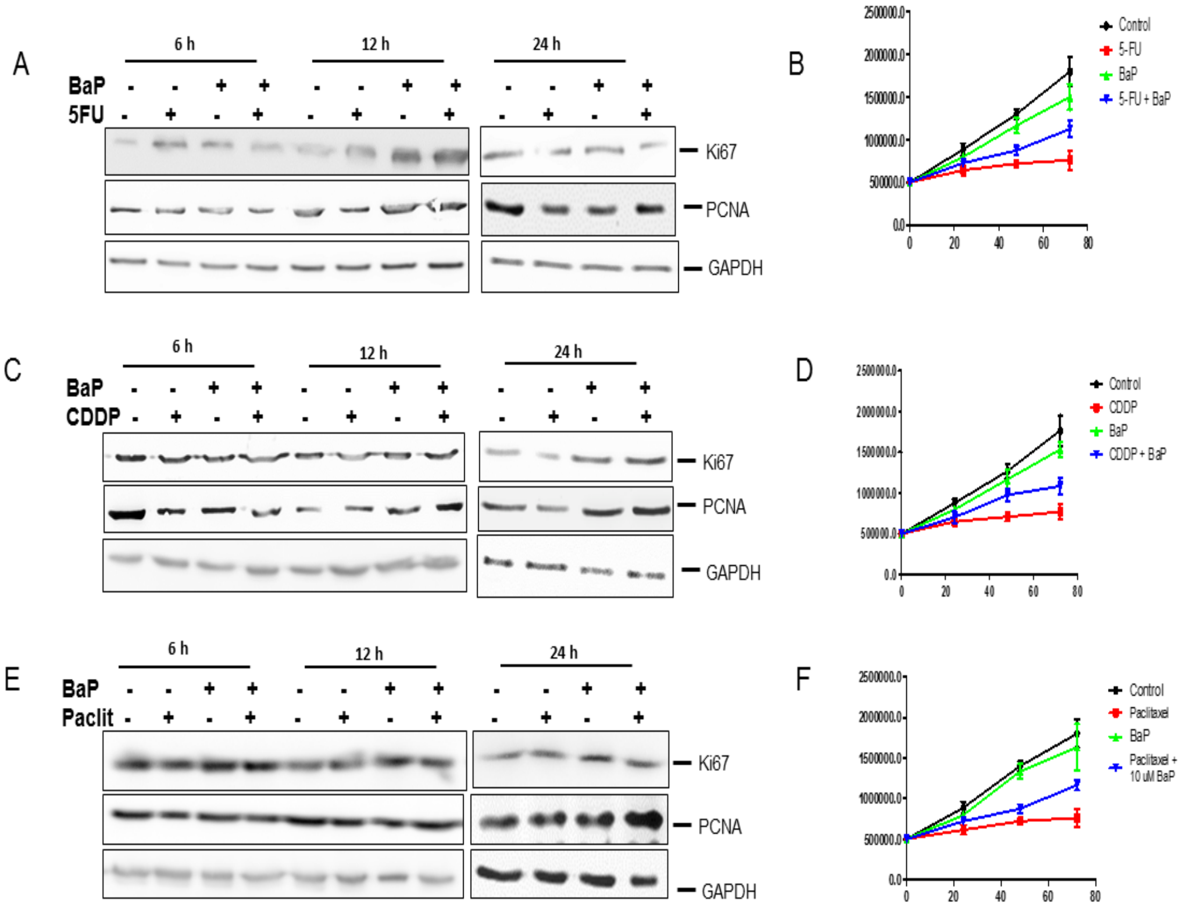

Supplementary Figure S2. Benzo-α-pyrene reverse the effect of cisplatin, 5-FU and paclitaxel on WHCO1 cell proliferation. WHCO1 cells (5 x 10^5) were plated in 6 well plates overnight. WHCO1 cells were then treated with 0.1% DMSO, 3.5 μM 5-FU, 4.2 μM cisplatin, 2 μM paclitaxel and 10 μM BaP for 6, 12 and 24 h. Cells were lysed with RIPA buffer and proteins quantified using the BCA protein quantification assay. GAPDH was used as a loading control. (A) Immunoblot analysis of WHCO1 cells treated with 5-FU and BaP using anti-Ki67 and anti-PCNA antibodies. (B) Cell proliferation was also monitored using cell counting method using the Countess automated cell counter after treatment of cells with 5-FU and BaP. (C) Immunoblot analysis of WHCO1 cells treated with cisplatin and BaP using anti-Ki67 and anti-PCNA antibodies. (D) Cell proliferation was also monitored using cell counting method using the Countess automated cell counter after treatment of cells with cisplatin and BaP. (E) Immunoblot analysis of WHCO1 cells treated with paclitaxel and BaP using anti-Ki67 and anti-PCNA antibodies. (F) Cell proliferation was also monitored using cell counting method using the Countess automated cell counter after treatment of cells with paclitaxel and BaP.

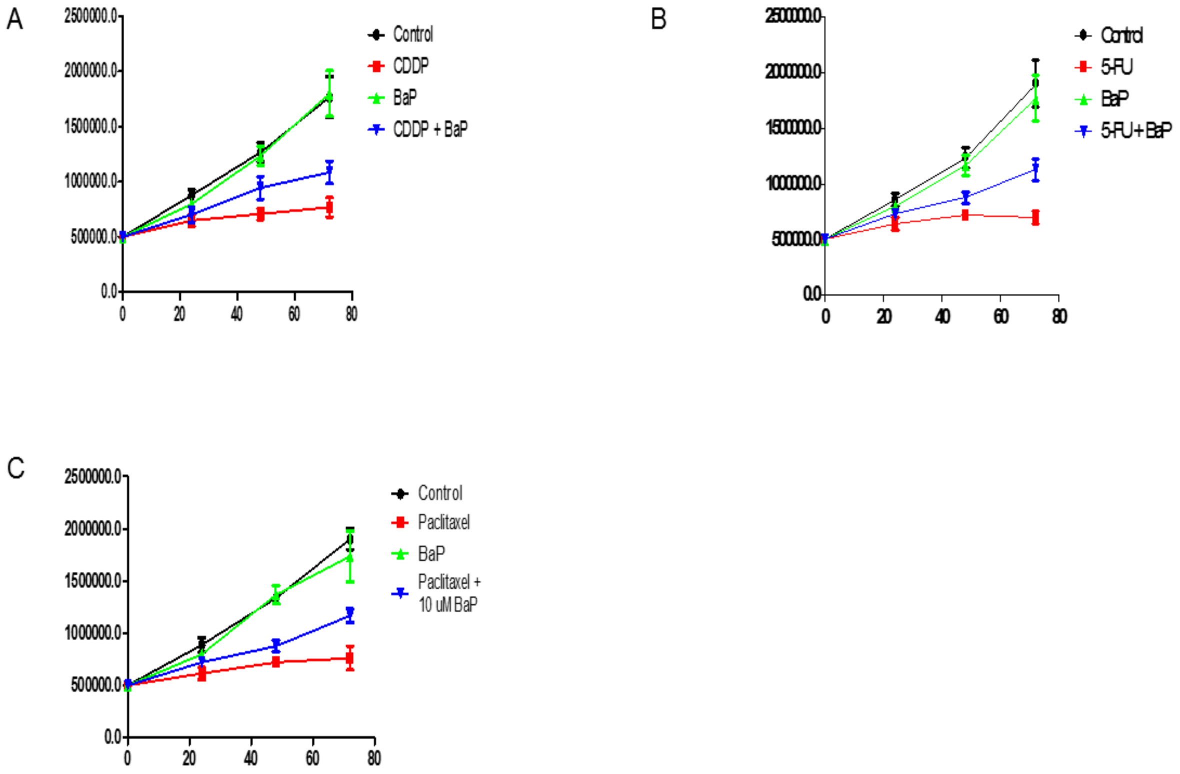

Supplementary Figure S3. Benzo-α-pyrene reverse the effect of cisplatin, 5-FU and paclitaxel on WHCO5 cell proliferation. WHCO5 cells (5 x 10^5) were plated in 6 well plates overnight. WHCO5 cells were then treated with 0.1% DMSO, 3.5 μM 5-FU, 4.2 μM cisplatin, 2 μM paclitaxel and 10 μM BaP for 6, 12 and 24 h. (A) Cell proliferation was monitored using cell counting method using the Countess automated cell counter after treatment of cells with cisplatin and BaP. (B) Cell proliferation was monitored using cell counting method using the Countess automated cell counter after treatment of cells with 5-fluorouracil and BaP. (C) Cell proliferation was monitored using cell counting method using the Countess automated cell counter after treatment of cells with paclitaxel and BaP.

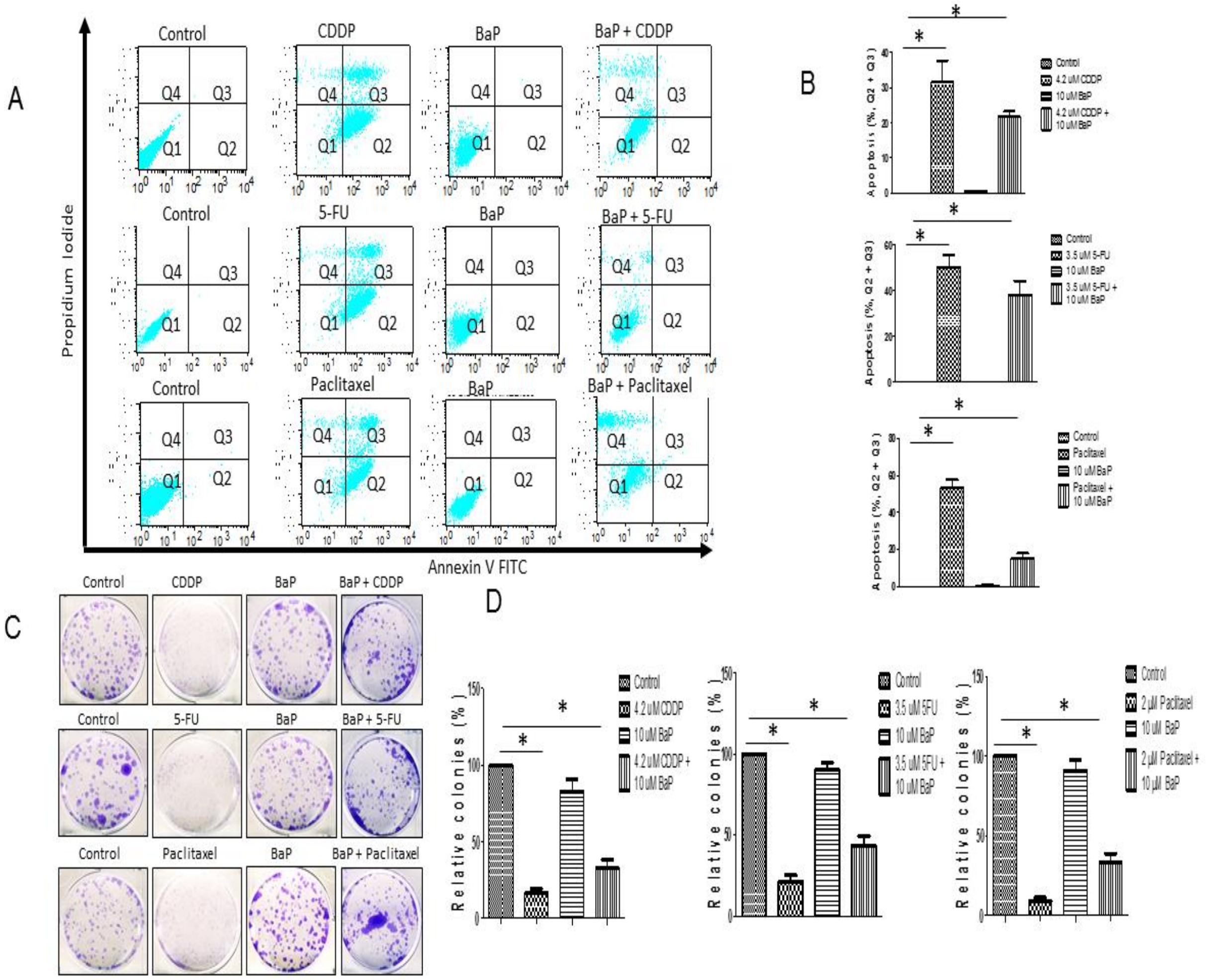

Supplementary Figure S4. Benzo-α-pyrene abrogates drug-induced- apoptosis and colony formation inhibition. (A) WHCO5 cells (3 x 10^5) were plated in 6 well plates overnight. WHCO5 cells were then treated with 0.1% DMSO, 4.2 μM CDDP, 3.5 μM 5-FU, 2 μM paclitaxel and 10 μM BaP for 24 h. Cells were double stained with Annexin V and Propidium Iodide and analyzed by flow cytometry to detect apoptosis. (B) Quantification of apoptosis after treatment of WHCO5 cells as described in (A) based on the percentage of cells in each quadrant (Q1, Q2, Q3, Q4). (C) WHCO5 cells (1 x 10^3) were plated in 6 well plates overnight. WHCO5 cells were then treated with 0.1% DMSO, 4.2 μM CDDP, 3.5 μM 5-FU, 2 μM paclitaxel and 10 μM BaP and incubated for 8 days. After 8 days colonies were fixed with 4% para-formaldehyde, stained with 0.1% crystal violet and counted. (D) Quantification of colonies after treatment of WHCO5 cells as described in (C). *$p < 0.05$.

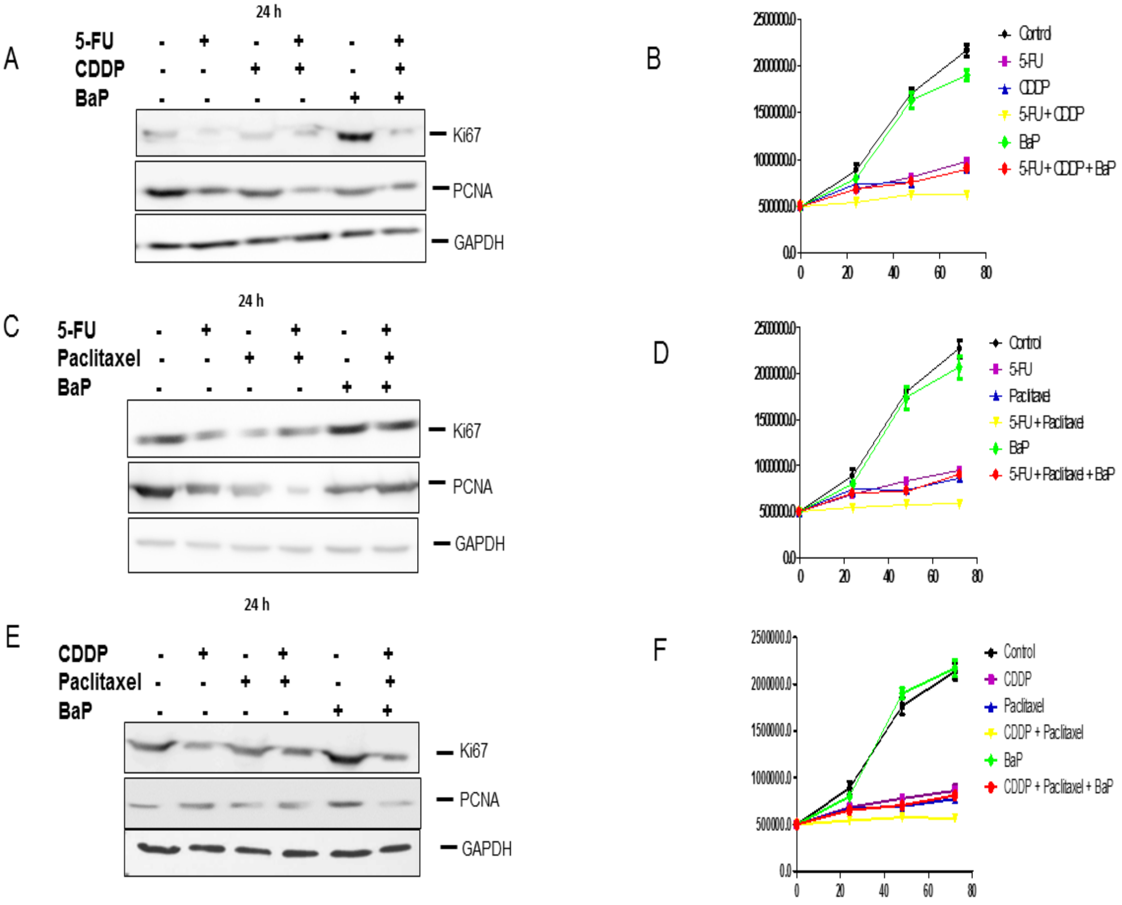

Supplementary Figure S5. Benzo-α-pyrene reverse the dual effects of cisplatin, 5-FU and paclitaxel on WHCO1 cell proliferation. WHCO1 cells (5 x 10^5) were plated in 6 well plates overnight. WHCO1 cells were then treated with 0.1% DMSO, 3.5 μM 5-FU, 4.2 μM cisplatin, 2 μM paclitaxel, 10 μM BaP and their combinations as indicated 24 h. Cells were lysed with RIPA buffer and proteins quantified using the BCA protein quantification assay. GAPDH was used as a loading control. (A) Immunoblot analysis of WHCO1 cells treated with cisplatin, 5-FU and BaP using anti-Ki67 and anti-PCNA antibodies. (B) Cell proliferation was also monitored using cell counting method using the Countess automated cell counter after treatment of cells with cisplatin, 5-FU and BaP. (C) Immunoblot analysis of WHCO1 cells treated with 5-FU, paclitaxel and BaP using anti-Ki67 and anti-PCNA antibodies. (D) Cell proliferation was also monitored using cell counting method using the Countess automated cell counter after treatment of cells with 5-FU, paclitaxel and BaP. (E) Immunoblot analysis of WHCO1 cells treated with cisplatin, paclitaxel and BaP using anti-Ki67 and anti-PCNA antibodies. (F) Cell proliferation was also monitored using cell counting method using the Countess automated cell counter after treatment of cells with cisplatin, paclitaxel and BaP.

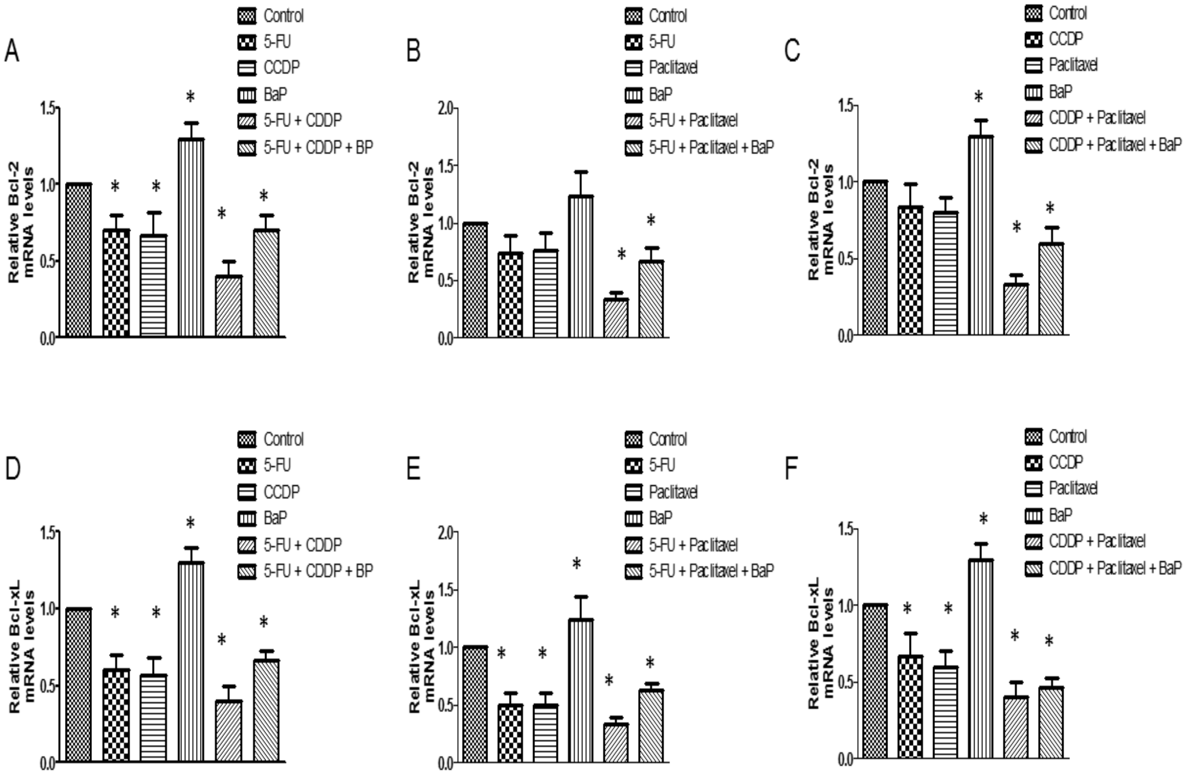

Supplementary Figure S6. Benzo-α-pyrene upregulates Bcl-2 and Bcl-xL expression in WHCO1 cancer cells. (A-F) Effect of BaP on WHCO1 cancer cell Bcl-2 and Bcl-xL gene expression in the presence of 0.1% DMSO, drugs, 10 μM BaP and their combinations for 24 h. Results are shown as an average of three independent experiments. *$p < 0.05$.

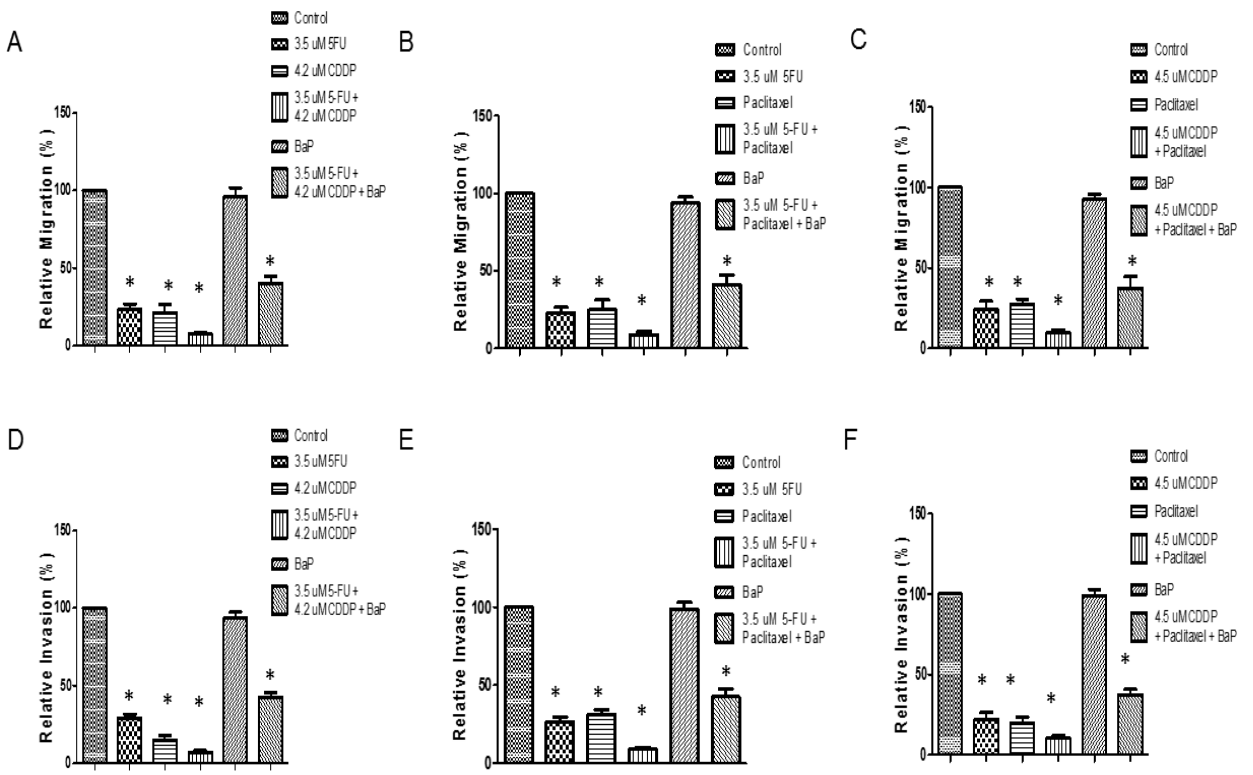

Supplementary Figure S7. Benzo-α-pyrene abrogates the effect of cisplatin, 5-FU, paclitaxel and their combinations on WHCO1 cell migration and invasion. Scratch wounds were made using a 200 ul pipette tip and cells were treated with 0.1% DMSO, 4.2 μM CDDP, 3.5 μM 5-FU, 2 μM, 10 μM BaP and their combinations 24 h. At indicated time points during incubation images of the scratch wounds were taken using a Phase Contrast inverted microscope (Olympus CKX41). (A-C) Quantification of the effect of BaP on WHCO1 cell migration in response to cisplatin, 5-FU, paclitaxel and their combinations. (D-F) Quantification of the effect of BaP on WHCO1 cell invasion in response to cisplatin, 5-FU, paclitaxel and their combinations. Results are shown as an average of three independent experiments. *$p < 0.05$.
